# Buttered Nostalgia: Feeding My Parents During #COVID19

**DOI:** 10.1177/02654075211012478

**Published:** 2021-05-04

**Authors:** Sandra L. Faulkner

**Affiliations:** Bowling Green State University, USA

**Keywords:** Critical family and interpersonal communication, family narratives, poetic inquiry, political difference, RDT (Relational Dialectics Theory)

## Abstract

The author uses poetic inquiry as CFIC (critical family and interpersonal communication) methodology to tell a story of cooking, cleaning, and caring for her elderly parents in the house she grew up in during the COVID-19 pandemic for 11 days in March 2020 when COVID-19 lockdowns began in the US. The piece is organized as a series of daily menus, lyric reflections, and narrative poems about family stories, family values, and the enactment of supportive behaviors that detail how a family deals with political differences, identity negotiation, and crisis. The author asks: (1) What does it mean to be a good daughter, and how is this complicated by discourses about the meaning of marriage?; (2) How does one reconcile family differences in political views and hold true to family and personal values?; and (3) How does one decide what obligations to focus on during a moment of personal and international crisis? The use of poetic inquiry shows how public cultural discourses influence private experience.

I began the project, *Buttered Nostalgia*, after spending 11 days with my elderly parents in March 2020 when COVID-19 lockdowns began in the US. On March 12, 2020, I drove south on I-75 from Ohio to Georgia with the fear that this would be the last time I ever saw my parents, because they were not in good health; my mother was 81 with a heart condition, and my father was 79 with COPD. During the visit, I cooked, cleaned, and cared for my parents while listening to family stories and spending hours doom scrolling on Facebook and news sources like NPR (National Public Radio) trying to convince my Fox News-watching-parents of the seriousness of COVID-19. I was terrified that I had brought them COVID, at the same time that I was grateful for the time I spent with them.

The poetic piece that follows tells this tale in poetry, menus, recipes, and images—language that best represents my experiences of and reflection on family stories and communication. I use poetic inquiry as CFIC (critical family and interpersonal communication) methodology to reflexively critique dominant discourses about family (Faulkner, 2016), and address the following questions: (1) What does it mean to be a good daughter, and how is this complicated by discourses about the meaning of marriage?; (2) How does one reconcile family differences in political views and hold true to family and personal values?; and (3) How does one decide what obligations—work, partnering, parenting, being a good daughter—to focus on during a moment of personal and international crisis?

*

## Friday, March the 13th

I stopped in Canton at my second parent’s house last night on my way to spend my spring break with my parents. I’ve known Charlotte and Terry since I was 5 years old. Their daughter was my best friend during elementary and high school, and they are my child’s chosen grandparents. We went to Longhorn’s Steakhouse when I arrived; I was nervous about eating out, but glad to drink a glass of wine and eat something off of a plate with silverware after driving for 10 hours. Usually, if I drive alone to my parent’s place, I stop at my older brother’s house in Lexington. But given COVID, I just wanted to get to my parents. Well, that and I didn’t want to listen to how COVID was a liberal conspiracy, and those who believe it are duped and stupid and communist and whatever other insults my libertarian gun-loving older brother would throw my way. Mostly, we have stopped talking about politics and focus on mutual interests such as our parents and music, but I didn’t want to risk it given what I had seen on his Facebook feed. Passive aggressive snark is our credo. I also didn’t call to arrange a visit with my younger brother who lives 20 miles from my parents much for the same reason. I have a better relationship with him, because he believes everyone has a right to their own opinion. The truth is that I’m not particularly close with either of my brothers. They don’t even call me on my birthday. Maybe I shouldn’t have stopped at Charlotte and Terry’s place, but I always visit them when I’m in the Atlanta area. They are family, too.

After breakfast, I call my mother. “Hey, Nanny! I’m going to stop by the store on my way to your place. Do you need anything?” I ask her even though I know what she is going to say—*Not that I can think of*. My parents act as if asking for anything is an imposition. Whenever I visit and do things for them, their profuse thanks makes me guilty. Guilty that I live 661 miles away and can’t help them with the everyday tasks that have become difficult since they are both disabled. Guilty that all I can do is cook and clean for them two to three times a year when I make time to visit. Guilty that every visit I am shocked at their decline; witnessing their aging is like going to a 30-year high school reunion and expecting to recognize everyone. When I visited at Christmas with my husband and daughter, we helped clean out the basement, and I cooked meals. *Thank you for all that you did*. Guilty that this has become their refrain when mundane tasks like cooking, cleaning, and keeping them company are the least I can do as a daughter.

“Milk? Eggs?” I know that I am going to stock up on staples, fresh fruit and vegetables regardless of what she tells me. My parents have been getting their groceries delivered for a few months now, because it is difficult for my dad to get to the store. Mom stopped driving sometime around 2005, and dad has been in a wheelchair since an aneurysm killed the veins in his right foot and leg necessitating an above the knee amputation 9 years ago.

*

The Super Target I find near Charlotte and Terry’s house is crowded and the shelves are bare from panic. I grab the last cart and swerve around a family in the entrance making a shopping list in my head. I sweat from anxiety as I stare down the other shoppers. *Does the woman who just sneezed by the beets have COVID? I wish that dude next to the bagged salad would move on already.* There are no canned beans on the shelf, and I snatch the last 20 pack of eggs getting too close to the bearded man studying the two bottles of half and half left. I don’t even try the paper good aisles, but I’m pleased to see there are still boxes of wine. I grab two plus some cans of sparkling wine and bags of chocolate truffles and peanut m&m’s. I intend to drink and eat my way through COVID.

When my cart with the double child seat is overflowing, I clumsily steer the beast to the checkout line with only five other carts waiting. I overhear conversations about schools shutting down and make a moral judgment about the couple in front of me sneezing and coughing. *Keep your nasty germs to yourself. Please don’t give me COVID.* My daughter was on spring break the week before, and she will be out for another week as the school district works out a plan.

*

I don’t hug my parents when I arrive. I have no idea whether I have COVID, so I intend to keep a 5-foot physical bubble between us the entire visit. We have never been a physically demonstrative family, so I hope this isn’t noted. I catch myself holding my breath whenever I hand food to my mom in her moss green recliner in the family room where she conducts all of her daily business.

After I put away groceries, I find some disinfectant in the kitchen cabinet under the sink and work on wiping down the counters, doorknobs, and light switches. They let me, but I can tell they think I’m overdoing it. Like when I called Nanny on Wednesday and asked if she wanted me to *not* drive down for spring break given the news about COVID. “Nanny, do you think I should stay here? I don’t think I’m infected, but I was just in Michigan giving that keynote presentation for Women’s History Month. I haven’t taught my classes in person for over a week, and I was careful not to get too close to anyone. But I’m in charge of an event on campus tonight.” My mother seemed confused by my call; it was clear that me not visiting because of COVID had not occurred to her because she didn’t think it was serious. “We always want to see you.”

From my spot on the loveseat next to Nanny’s recliner, I go online to read my email and start reading her news from my Facebook feed being sure to cite information from the CDC and articles that rely on science. I hope that repeating the horror that is unfolding will penetrate. Convincing my parents, who watch FOX news—the station that helped politicize public health and downplay this virus—that COVID19 is no joke is going to take some work. I see a message that my younger brother posted on Facebook complaining that America has lost its mind with COVID restrictions. I hope that he won’t come and visit, though I usually see him when I’m in town. It’s bad enough that I’m here. I don’t want anyone else in this house potentially giving my parents COVID. I open an email from my provost and find out that the university will be online for a week after spring break. I decide to worry about teaching prep later.

## Day 2, March 14th

The clock shop is tucked in the back of a flea market antique shop about 25 miles from the house that I was born and raised in. Dad drives us in the old rusty-white Ford pickup truck he got from my younger brother’s business. He had it refitted with a wheelchair lift and a left-footed gas pedal. I don’t want to be going anywhere, but he’s been asking me since I arrived if I would go with him to pick up the repaired ship’s clock that has hung in the family room my whole life. Their house sits on an acre of land in a subdivision that was built in the 1970s and early 1980s, filled with brick ranch and two-story split-level houses. Some of the original families, like my parents, still live here, though many left in the 80’s in a wave of white flight as the county became predominantly African American. At least, I convinced him that going to Red Lobster for lunch was not a good idea. I keep repeating to him and my mother they’re at risk for COVID, especially given their age and health conditions. “Besides, I bought all of this food I need to cook.”

On the way home, I have him take me by the liquor store to get a handle of Maker’s Mark. The boxes of wine aren’t going to do it, as I intend to drink my way through this week and my increasing anxiety. Luckily, because he is friendly with the owner of the store, we pull up by the curb and one of the store clerks brings us what Dad has written on a piece of paper and loads it into the truck. I’m pleased that Dad is still a great tipper. “Booze is a luxury that I don’t need, so if I can afford it, I can tip.”

When we return home, I take a walk/run on the nature path next to the subdivision even though my left heel spur pain has flared up. I’m pretending to balance my vices—drinking and sugar—with healthy ways of dealing with stress like cooking, running, and writing poetry. Being outside smelling the pine trees and picking up pieces of granite brings back nostalgic glimpses of growing up here. Being outside is a reprieve from being a daughter, a parent, a teacher, and a spouse.

In the afternoon, I tackle cleaning Dad’s room. I dust around the shot gun propped up against one of the windowsills. I’m afraid to move it, so I carefully run the vacuum cleaner in a wide arc around it. *I hope Dad never gets too depressed about health issues. He wouldn’t use this, would he?* There is a gun in most every room of this house, which I note when pulling out the kitchen drawer looking for batteries and when looking for stamps in the writing desk. I don’t bother to open the glovebox in the truck. As I put away laundry, I see a train set from my dad’s childhood on top of his dresser. It seems we are both riding in a boxcar of childhood memories.

*

### Dinner menu

[I riffle around in the deep freeze to see if I can find some protein to make with the fresh veggies from Target. Over Christmas, I had cleaned it out, throwing out three-year-old desiccated bread. I score when I find some chicken breasts still in date].

Puff pastry stuffed with chicken breast dry-rubbed with poultry
seasoning,
wilted spinach with balsamic vinegar and olive oil, red grapes,
and cheddar cheese,
served with a side of roasted broccoli sprinkled with parmesan,
sea salt, and lemon juice
2 glasses of boxed Shiraz followed by two fingers of Dad’s
20-year-old Jameson

## Menu, Sunday, March 15th

Grilled Sablefish with a side of Gingered Bok Choy
Lemon Bars with a side of Maker’s Mark

Dad complains that I didn’t cook the sable fish enough and puts his plate in the microwave. “Dad, you’ll ruin it! It’s cooked.” I yelled.

“I’ve cooked this before. I know how it’s done.” He said and added another minute to the timer. I whisper to Nanny that *he is ruining the fish, it is soft and oily and does not become firm and dry when cooked*, and then I post about it in a small private Facebook group I belong to for foodies called *Eat Share Eat*. This group originated in Pittsburgh but is now composed of friends of original members from professional chefs to amateur bakers like me. What we have in common is a love of food, drinks, and liberal politics:

I made it to my folk’s place in Atlanta on Thursday. They are 79 and 81 and in poor health. I have been cleaning and cooking for them. I have made some of the best food. (Yesterday: puff pastry with chicken in poultry seasoning, spinach with balsamic and olive oil, red grapes, and cheddar). Tonight, I cooked sablefish. I made it medium as it should be cooked. He bitched that it was undercooked and put it in the microwave until it was ruined. HELP!!!!

**Figure 1.**
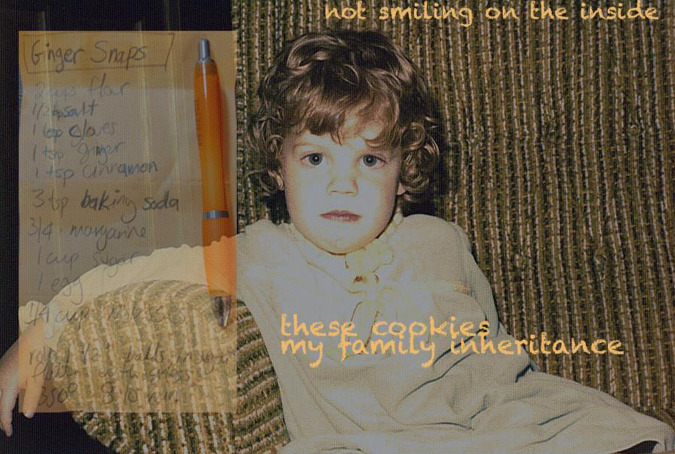
Giant Ginger Snaps

A member of the group tells me to be gentle as he describes caring for his aging mother. I make a mental note to be kinder, though I learned all of my kitchen irks and quirks from my father. We know what we want and we the way we want it rules our kitchen and our kitchen etiquette. *Maybe Dad doesn’t like sharing his kitchen with me* (see Figure 1).

*

Giant Ginger Snaps^1^

My ex texts to ask about her gazpacho
the taste is too sharp, the afterbite too acrid-
I advise her to try a teaspoon of honey,
though I am always less precise
tossing in garlic until it smells right
tasting my way through a recipe
like my dad taught me
when I was his commis chef.
We cook by intuition and feel
follow our inner hunger,
he liked the onion chopped in big pieces
so you could see it in the finished dish
have something substantial to chew,
no disguising the bite of allium-
Once she, or some other ex, called me
a Kitchen Nazi because I know
what I want in the kitchen
and I won’t back down,
my sense of self too strong
from the time I learned not to smile
for the camera because I’m
not smiling on the inside so why lie?
I mail her a box of ginger snaps every Christmas
because they are her favorite of the recipes
I copied down by hand from my parent’s cookbook stash,
my inheritance from a line of family cooks.

## March 16th, COVID Day 4

A question posted on my foodie Facebook group today: What’s the most unexpected thing you’ve found in your kitchen cupboards when you did your “how long can we survive without going out” stock take?

What I find in my parent’s pantry (A list poem):

Two cases of Campbell’s Beef Noodle Soup,
8 bottles of low-sodium ketchup,
a case of Italian tuna in extra virgin olive oil,
4 jars of Hellman’s mayonnaise,
enough pasta to open a pop-up restaurant,
4 cans of smoked kipper snacks,
3 tubs of Betty Crocker cream cheese frosting,
2 cans of pumpkin mixed in with 4 cans of pineapple
chunks,
6 bottles of salad dressing (they still love Thousand
Island),
3 jars of crunchy natural peanut butter and 1 jar of hot peanut
butter,
8 jars of low-sodium beef bouillon, 1 jar of low-sodium chicken
bouillon,
1 jar of vegetable bouillon with enough sodium to
preserve
the entire neighborhood, and 3 boxes of roach motels

When I take a break from cabinet organization and check my email, I find out that my university will be online for the remainder of spring semester. I resent the email after email after email that I receive from my provost and dean and school director and university president, because I have to transform my in-person classes to online classes over my now canceled spring break. I ignore Facebook posts by liberal friends who are unfriending and cutting out people and family members who support Trump. I’m grateful that my BFF in Gerontology messages me daily to see how everything is going. We commiserate over conservative family members and doing the right thing and how we can’t just stop talking to them. She tells me that I’m a good daughter. *Damn it. I just want to be with my parents. And damn it that I have to break my vow to never teach online.*

*

Still Life with Dad’s Chocolate Bowl^2^

The salad bowl/a wedding present/the bowl with a pewter band/the
stash of chocolate/always Hershey and always King Size/the bowl tucked
in the cupboard by the refrigerator/the bowl with odds and ends rests on
a shelf/ beside the cocktail shaker and boxes of broth/too high to see
inside/small hands stretch up in search/smear the grease of childhood on
the rim/the bowl holds domestic detritus/never the acid of dressing/but
flashlights, batteries, and books of restaurant matches/49 years in the
same place/scenes of domestic reckoning by the bowl/the time Mom threw a
pack of hotdogs at Dad/her anger thudded against his chest/and fell to
the floor by his feet/his arms and hands glued to his sides/kids stunned
into quiet/the uncharacteristic turn of passive aggressiveness/by the
cupboard/checking for chocolate/the bowl with the sweets and the
secrets/menthol cigarettes before he quit/if you pilfered one or two out
to smoke with a friend behind a car in the cul-de-sac/no one noticed the
theft/the bowl was always full of jumble/like the noise of a full
house/sometimes foil packs of airline peanuts out of the
briefcase/locked with a brass key/black government issue ball-point pens
that skip on paper/kids still creak open the door/their grown hands
search for treats/in the bowl with bolts and odd screws to machines no
one remembers

## March 17th, Lunch Day 5

### Eat Share Eat Facebook post (see Figure 2)

**Figure 2.**
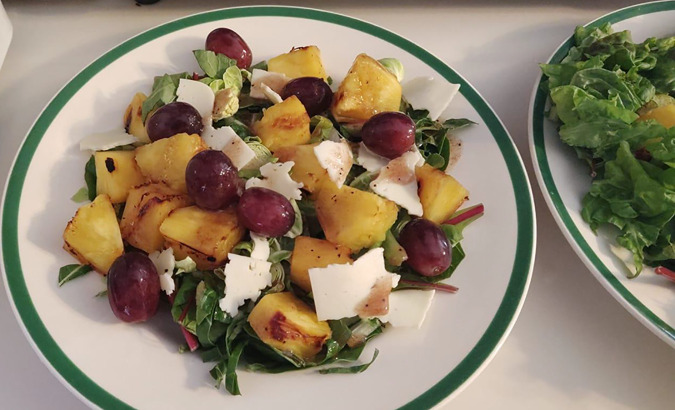
March 17th, Lunch Day 5.

I made mom grilled pineapple salad with shaved Brussel sprouts, bok choy, field greens, Havarti cheese, red grapes and marionberry dressing and served it on a kelly-green rimmed salad plate. Dad ate two Tastykake fried apple pies. He chased it down with a fistful of Doritos. When I was a child, we did not have junk food in the house. #feedingelderlyparents

After lunch, I scroll through more COVID news, reading mom the serious facts, as well as sharing the funny memes about toilet paper. Dad has always kept a large stash and even carries a roll in his vehicles for the just in case. I only counted eight rolls in their inventory, which is not enough given there is no toilet paper after everyone’s panic buying and hoarding. I’m grateful that my case of recycled bamboo paper was delivered right before I left and feel smug that actualizing my dad’s lessons means I have a 4-month supply. Nanny tells me that my younger brother has a case and can bring some over and leave it by the door in an emergency. “He has been sick with pneumonia for two weeks, so he said he will see you next time.” I’m relieved. I find out months later that he thought he had COVID, so he stayed away.

We talk about one of her best friends who had cancer. I try and use this as an in to talk about her COVID risk:

Nanny: I am worried about Linda. She is in remission, but the treatments destroyed her immune system.

Sandra: *You* have a heart condition and *dad* has lung problems. *You* are the vulnerable, too.

Nanny: I know. (She doesn’t sound convinced).

Sandra: Trump has handled this whole thing poorly.

Nanny: All politicians disgust me.

## Wednesday, March 18th

#feedingmyparents Day 6 COVID19

Sandra: Are you having Doritos for lunch?

Dad: Yes. I ate an apple first.

Sandra: They cancel one another out. I suppose Doritos are part of the cheese food group.

**Figure 3.**
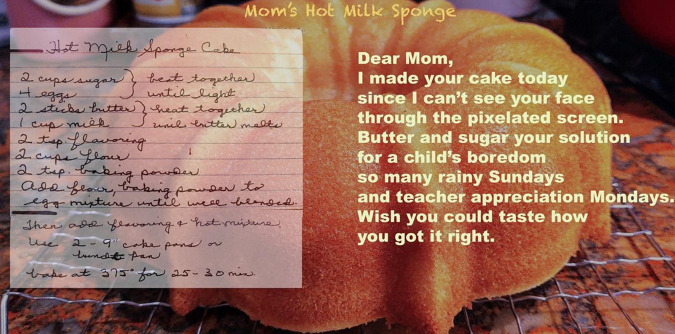
Mom’s Hot Milk Sponge.

Mom and I eat salad and hummus. I give us the rest of the lemon bars I made on Monday for dessert. When I lived here, we always had homemade dessert, so I made these bars a few days ago knowing they were one of her favorites. Dad doesn’t make dessert, though he does all of the cooking for them. So many Sundays I would be bored and want to bake, and she was a patient teacher (see Figure 3). Not like me with my daughter, Mimi—I like being in the kitchen alone and don’t like to talk while working. And I’m impatient with those who cannot read my mind.

I find out that my Uncle Ed who lives in Kentucky is coming for a visit tomorrow. *I finally convinced my parents that COVID is real, and now he’s visiting?* He still goes to the grocery store every day. Nanny tells Dad to call him. Dad doesn’t directly tell him *not* to come, and Ed chooses *not* to pick up on the subtlety. Nanny explains that he has never been good at telling his brother no, and because he is 9 years older, I sometimes wonder if he plays a bit of a father role in my Uncle’s life.

*

### Dinner #feedingmyparents

So, my mother and I had hamburgers with a side of roasted broccoli. Dad has opted for the bag of Cheetos.

And for dessert? He ate two Mr. Goodbars.

Someone in the foodie group asks me if my dad eats hamburgers. I respond:

He will. He cooked them for my mother and me, but he doesn’t eat until late. He also drinks a few beers, so then that kills his appetite. He decided that he would rather have the Cheetos. This man is not the one who fed me when I lived in this house.

Another member asks me how old my parents are and confesses:

My mom eats ice cream 3x a day (92 with Parkinson’s). We say fuck it!

*

I wake up with night sweats, and as usual, whenever I’m awake the word COVID occupies my entire being. The word flashes in my head in bold print lettering, first and last thing and most of the time in between. Being perimenopausal and trying to sleep in this house when Dad turns on the heat in the spring in Atlanta when there is 50% humidity is like wearing a parka in a sauna. I open the window above the bed and turn on the ceiling fan full blast hoping I can suck the heat out. I think about what I will cook tomorrow and make a list of other household tasks to be done.

*

Mom’s Hot Milk Sponge^3^

Dear Mom,
I made your cake today
since I can’t see your face
through the pixelated screen.
Butter and sugar your solution
for a child’s boredom
so many rainy Sundays
and teach appreciation Mondays.
Wish you could taste how
you got it right.

## Thursday, March 19th

After lunch, Dad’s eyes stop working; he has these visual migraines more often since he lost his leg. He rests in the family room on his torn and taped brown leather recliner with the lights out. I remember lying in this room on an avocado green and orange plaid cloth loveseat when I was in elementary school, burning with fever, mom taking my temperature every 5 minutes and replacing the wet washcloth on my forehead. *Don’t we all need some mothering now?* I decide that I’m going to stay longer since classes are online. Mimi keeps telling me how much she misses me when I call, but I’m certain that I won’t get to spend time with my parents like this ever again. I am stretched like a rubber band across four state lines, and while work can be done anywhere, being a good daughter and being a good mother and partner are rooted in physical spaces 661 miles apart.

*

Mother is^4^
a verb: to Mother is not biology
or sex
not tied to body parts
but action
and oh how I want to be Mothered now;
they said rest when you become a Mother
but there is only touch
and holding one another
so touch me like storied silk;
think of how white tail rabbits
weave a nest out of grass and their own hair
avoid the spot during daylight
to not alert the neighborhood dogs
feeding their kittens in the crepuscular light;
let’s make a poultice out of silk
and rage, stir the pot of trouble bubbling
with the spoon of the not-quite Mothered;
I need this dream of our Mother
how she writes the world with a breath
of liquid wrath suspended in a candy bubble;
she swaddles her anger and places it
in a nest of rabbits, wakes up to find
a bouquet of roses in the mailbox;
let Mother be the knot of silk
giving you something to hold
and tie your pain to the knotted places;
Mother knits your clothes in silk
as soft as rabbit fur
so you can breathe exhale
run so fast they can’t hunt you
as you thump your displeasure;
we can’t drink from a dry wild, Mother.

## COVID Day 8, Friday, March 20th

My Uncle and I take a walk with Jabber, one of his rescue greyhounds who made the trip with him. Though, I’m still irritated he came to visit, it’s good to see him. I remember how he visited me in England in 1991 when I was studying abroad in a small town 180 km north of London. He took a train and a cab from London just to pick me up and take me out for Indian food. We ate and talked, and then he took the train back to London and his business trip. I forgive him for visiting when I realize that we both had to make this trip—*who knows how much longer Mom and Dad are going to be alive*. As we walk, we commiserate about my father, his generosity and his stubbornness.

“Your father has been telling me how to do things I’ve been doing for almost 70 years. I don’t remember him being this way.” My Uncle said. He moves Jabber to the side so some people can pass.

“I know. I think it’s gotten worse. He just knows how to do things!” What I don’t tell him is that I’m more like my father than my mother. Just this morning he reloaded the dishwasher after I had loaded it. And when I am home, I do the same thing. After all, I know how best to position the dishes to maximize the spray arm’s reach.

*

Dad asks me why I don’t come out from my mom’s bedroom—my make-shift office—at 5 pm for my nightly cocktail. He and my Uncle want me to help drag Christmas dishes from the kitchen to the basement, but I’m furiously trying to record a PowerPoint lecture. I have less than a week to get two previously face to face classes online. This is a race I didn’t want to run, especially while on “break.” I pour a double Makers Mark over some ice and lock myself in my mother’s bedroom to create online lectures about family communication.

### Dinner menu

For me, a Bagel Dog from the freezer served with Dijon mustard to soak up the class prep bourbon
For my mother, Grilled Tofu with Jerk Seasoning and Roasted Cauliflower

*

Phantom Pain^5^

You can’t re-member the advice
you lost by staying in bed,
not getting up to write it down,
so you sit at the kitchen counter
stare at the box of Spanish wine, the wilted
lilies
in a chipped vase beside your kid’s orchestra practice
log,
and try drowning in this dream to remember your
father,
though he is still physically present,
his silent lessons dim in the light of your
childhood,
the flickers of memory make monsters
that stroke your fear, shriek at your nostalgia
and cackle when you can’t recall
the lines of poetry that summon your feelings,
so you grasp at the patches of memory:
the taste of perfection in his over-easy eggs,
not a speck of brown tainting the edges;
the smell of his bitter coffee that can keep you
awake
for weeks, the flavor over-pronounced, too
intense;
his signature smirk with only a slight curve of upper
lip
when something is more than okay;
all of the animals and kids know
his whispers only they can hear;
how he can rig anything to work again with just-in-case
parts
kept in old medicine bottles and baby food
jars;
the way he used his white-collar hands on
weekends
fixing transmissions and changing oil for the Church bus
ministry,
and why he quit being a Deacon
when the pastor cried too many Blacks at
Church,
and did not move when White Flight
arrived in our Suburban Atlanta neighborhood;
the quiet paying of neighbor’s bills,
the time spent scrubbing the week’s grease off pots and
pans;
always giving you $.50 cents instead of the $.25
offered
to get him a beer out of the basement fridge;
when he once told you that *Life is a series of
disappointments.*
*And then you die.* And still, he showed up with
rolled up sleeves;
You have already started mourning
and give in to the phantom pain,
so in meditation you can’t picture a garden
like the guru instructs,
instead you go back to the woods
where if you hike in far enough,
you can bathe in the Sweetgum’s shadow,
breathe in the medicinal pine,
to focus on the whine of work,
and hear your too-human sounds.

## Saturday, March 21st

**Figure 4.**
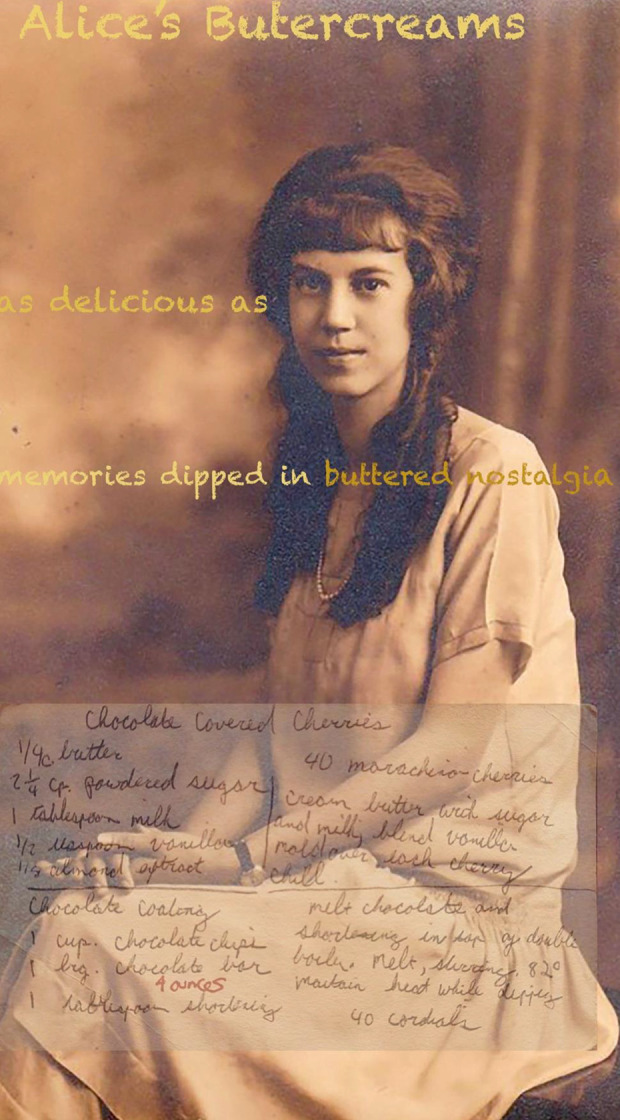
Alice’s Buttercreams.

Dad eats a bowl of ice cream for lunch, and I feed leftover jerk tofu to my Uncle and Nanny. Ed has been interested in family genealogy for a while and asks me to guess which one of three family stories he remembers hearing as a child is true (see Figure 4). I guess wrong.

Nanny and I watch the baby animal marathon on Animal Planet. I have my laptop open half-heartedly working on my classes. Dad comes into the family room and changes the channel to Fox News. “We don’t want to watch that.” Nanny changes the channel back to the baby animals. *She has been paying attention to me. She knows I hate Fox News! Maybe all of my talk about COVID is working. I have not backed off criticizing Trump, which I thought was annoying her as she usually deflects with a comment about how all politicians are bad.* We reminisce about things that happened when I was in high school and watch puppies, feline kittens, ferrets, hamsters, and rabbit kittens scurry, slide, and skitter their way into a sheltered world.

### Dinner

Low Sodium Beef Brisket with Horseradish Sauce
Egg Noodles with Pasta Sprinkle
Bok Choy with Leftover Green Beans

Sandra: The meat is tender, Dad. Josh doesn’t eat beef anymore, but every 6 months I like a filet.

Dad: As long as you eat a balanced diet. Of course, I should eat vegetables. I just eat meat and potatoes. But at this age, I don’t give a shit.

Sandra: You are a mutant, so it doesn’t seem to make a difference.

Dad: Your mother gets upset, because she watches what she eats. My triglycerides were high a few years ago. But just now, my blood work was good.

Dad: Where is the grain? I need to cut across it.

*

Alice’s Buttercreams^6^

My Uncle tells me family stories
asks me to guess which one is true
though stories don’t fact-check,
their science more like the art of truth-telling,
turning the family into what and who
and where and how we need to be:
Did Grandma Maude and family own the land
where Philadelphia City Hall now stands?
or
Was Nipper, the RCA Victor dog, our family
canine posed for the picture by the gramophone?
or
Where did my Great Uncle get his money for the
boats
and the shore house on a petty officer’s salary?
I study the pictures of him with handsome men
and like where my mind wanders
cooking up a saucy sweet family secret.
or
Was my Great Uncle Hemerley’s Smith & Wesson revolver
claimed
from the body of a dead Mexican soldier in the Spanish Civil
War?
or
Are we related to the (rich) Habbersetts of Scrapple
fame
from Middleton, PA? Is this why my parents made me eat
it?
Did my Dad pay my Uncle $5 to eat a batch of his
mother
Alice’s buttercreams that she was going to take to
church?
Or was it $.25 for a box of commercially produced
candies?
And are they as good as the memories?
I ask my mom for the recipe, and she confesses
she threw it out because the actual taste is not
as delicious as memories dipped in buttered
nostalgia.
or
What if I tell you I have the recipe for Alice’s
Buttercreams?
I got it from a college roommate and make them for my
Dad,
and my kid’s classroom Valentine parties,
though they contain cherries and sometimes,
bourbon,
and use whatever chocolate I have in my pantry
because this is how you turn the quotidian into family
myth.

## Sunday, March 22nd

After my Uncle leaves, Nanny and I watch my church’s service on Facebook Live. I don’t sing during the hymns, because I’m not standing in a sanctuary with Josh by my side. *God, I miss him*. I tell Nanny how he often chooses this point in the service to leave and get a cup of bad church coffee. Things are casual at the Unitarian Universalist Congregation—I often knit during service—and more than half of the members are atheists like me. I have Nanny watch, because I want to show her that I’m part of a faith community, even if it is nothing like her Episcopal upbringing and my early years in our Baptist Church. “I loved the hymns we sang as Episcopalians, but I can’t sing anymore. I just sound croaky.” Nanny said.

I check the news after the Sunday reprieve to find out that the Ohio governor, Mike DeWine, and the health director, Dr. Amy Acton, have declared a stay-at-home order which will begin on Monday at 11:59 PM. *Should I leave before then? What if I can’t get back into the state? Should I stay? Should I go?* I feel torn, but Nanny tells me I should go home to Josh and Mimi. I was planning on staying through Friday, but I decide to leave on Tuesday morning.

I put on my shoes to take a walk, and see that Dad got the riding mower out. He buckles himself into the mower with a canvas strap and places an extra quart of gas in an aluminum water bottle just in case he runs out. He would be stuck down the yard, as my mother can’t walk on uneven terrain. I skip my walk and work on picking up sticks to help Dad. And by sticks, I mean some branches as big as small trees. I drag them around the house to the old burn pile on the back rocks, the ½ acre of exposed granite at the rear of the property. We work all afternoon, and I see on my Garmin that I walked 3 miles. Neither of us is good at being idle.

For lunch, I heat up a can of mock turtle soup, and Dad eats crackers with port wine cheese spread. I lock myself in Nanny’s bedroom and message the owner of my hair salon to buy gift cards. I saw her Facebook post about salon closings, and since I just got paid, it’s the least I can do. *Who knows how long the business will be closed?*

### Dinner

Two old-fashioned cocktails made with maraschino cherries from the pantry, the blood orange Nanny got in her fruit of the month club, and my stash of Maker’s Mark served with ½ pound of peanut m&m’s

The menu above was *my* dinner. I’ve been judgmental about my father’s diet, but before I leave, I will have drunk 1 liter of Maker’s Mark, five glasses of Malbec from a box, four cans of sparkling wine, and eaten 2 pounds of m&m’s, and one box of S’more Girl Scout Cookies.

### Dinner #2

Dad makes baked sweet onions with bouillon, a dish he learned in Boy Scouts. You peel a sweet onion, tuck a bouillon cube inside with a generous amount of butter and wrap it in aluminum foil. Roast in the oven or in a fire pit until the seasoning has made a bold sauce, and the onion is soft as spring skin.
He serves the onions with pan-roasted salmon and halibut and a side of baked sweet potatoes.

## Monday, March 23rd, Family Photos

**Figure 5.**
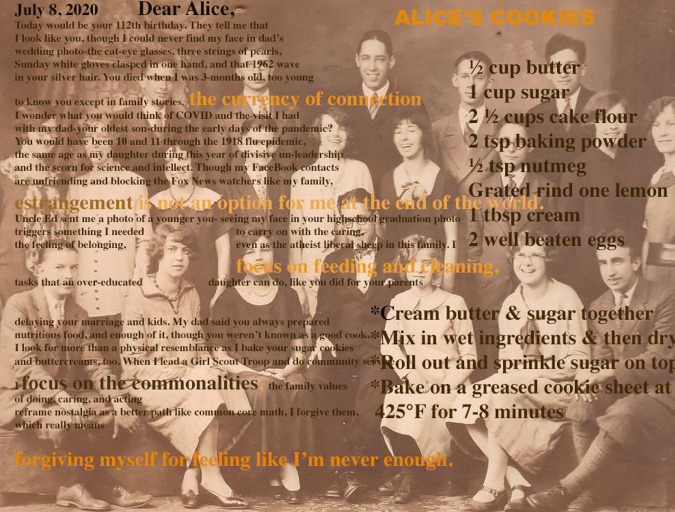
Dear Alice.

My Uncle sends me an email telling me that he showed his wife a photo of his mother’s high school graduation. “See if you can find Alice.” Amy immediately picked her out of the sepia sea of faces because I look like her. My mother has told me before that I favor Alice, but the only photograph I’ve seen is one from my parent’s wedding of an older woman in a church dress with black cat-eye glasses. I find my face in that old photograph not realizing how much I needed to see myself in family (see Figure 5).

July 8, 2020 Dear Alice^7^
Today would be your 112th birthday. They tell me that
I look like you, though I could never find my face in
dad’s
wedding photo-the cat-eye glasses, three strings of
pearls,
Sunday white gloves clasped in one hand, and that 1962
wave
in your silver hair. You died when I was 3-months old, too
young
to know you except in family stories, *the currency of
connection*.
I wonder what you would think of COVID and the visit I
had
with my dad-your oldest son-during the early days of the
pandemic?
You would have been 10 and 11 through the 1918 flu
epidemic,
the same age as my daughter during this year of divisive
un-leadership
and the scorn for science and intellect. Though my Facebook
contacts
are unfriending and blocking the Fox News watchers like my
family,
*estrangement is not an option for me at the end of the
world*.
Uncle Ed sent me a photo of a younger you- seeing my face in
your
high school graduation photo triggers something I
needed
to carry on with the caring, the feeling of belonging, even
as
the atheist liberal sheep in this family. I *focus on feeding
and cleaning*,
tasks that an over-educated daughter can do, like you did for your
parents
delaying your marriage and kids. My dad said you always
prepared
nutritious food, and enough of it, though you weren’t known as a
good cook.
I look for more than a physical resemblance as I bake your sugar
cookies
and buttercreams, too. When I lead a Girl Scout Troop and do
community service
I *focus on the commonalities*, the family values of
doing, caring, and acting,
reframe nostalgia as a better path like common core math, I forgive
them,
which really means *forgiving myself for feeling like I’m
never enough*.

## Tuesday, March 24, 2020

I take a shower at 4:45 am to be ready to leave before the crack of dawn. It’s a long drive, and I’m not sure what the roads will be like given that I-75 is usually one traffic jam after another. Though, given the lockdowns who knows? Nanny had a difficult time getting up, because we had stayed up late sorting her yarn stash for me to take home; the bags are piled in the back of my Rav4. A snack bag with a sandwich and apples is on the passenger side seat. I plan to only stop for gas. I will pee on the side of the road, so that I don’t have to go inside anywhere. I will use latex gloves to pump gas and sanitize my hands every 100 miles.

At 5:15 am I stand in the kitchen with my keys ready. Nanny and Dad blink back sleep and look at me. I tell them that I won’t hug them goodbye. “I just want to be safe.” Though, the truth is that I don’t want to cry. And if I hug them, there is no way I can pick up my to-go coffee and walk to my car and drive away. *This may be the last time I ever see them. God, I wish this was hyperbole.*

**Stay home Ohio. Stop the spread of COVID 19.**

**Figure 6.**
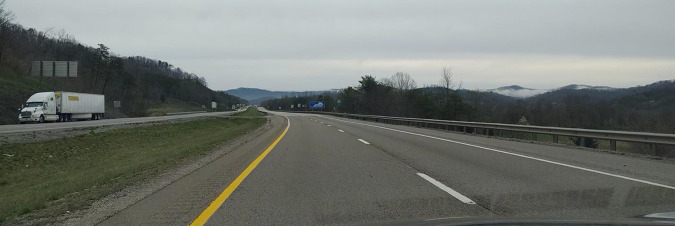
I-75.

This is the electronic sign that greets me when I enter back into Ohio. A flashing red warning that life has altered more than my ability to imagine it. The interstate has been empty in a post-apocalyptic way. This may very well be the apocalypse. Driving north on I-75 away from my parents is the hardest thing I’ve done. Maybe ever (see Figure 6).

Which of the following stories is true?

**One**

I make it to Chattanooga before I turn around. I cry the 2.5 hours it takes me to end up back in my parent’s driveway making driving difficult. I run out of paper napkins and have to use my shirt sleeves to wipe away my salty snot. I hug my parents and tell them that I love them. After I drink another cup of coffee, I get back into the Rav4 and begin the trip home for the second time.

**Two**

I make it 2 miles to the interstate and turn around. I’m going to stay for another week.

**Three**

When I see the COVID sign as I enter Ohio, I finally let myself weep. I call my mother to let her know I’m home, and she tells me that I made the right decision. “I’m glad you’re back with Josh and Mimi where you need to be.” She released me from guilt like a good mother. I could have stayed apart from my spouse a bit longer, but not my kid. *Being mother is the action that lets us do the difficult things, lets us endure the inevitable ruptures, lets us break apart and mend ourselves, only to be broken again and again.*

## Method

CFIC focuses on issues of power, resistance, critique, and transformation of the status quo, collapsing the false binary of public/private, and highlighting author reflexivity (Suter, 2018). I used poetic inquiry as a CFIC method because of its ability to show dialectics and tensions in family life, and its potential as feminist embodied inquiry (Faulkner, 2016, 2018). Poetic inquiry is a form of Arts-Based Research that highlights the aesthetics of personal experience, focuses on embodiment and participatory measures, and uses artistic forms to meld scientific and humanistic understandings of relationships. The use of poetry as a form of storytelling and feminist work provides CFIC researchers a way to show dialectics and tensions in family life, provide alternatives to the status quo by critiquing taken-for-granted social structures and assumptions about relational life (Faulkner, 2021). “Poetry promises to return researchers back to the body in order to demonstrate how our theories arise out of embodied experience” (Faulkner, 2017b, p. 214). Poetry offers a way to tell embodied evocative stories that engage academic and nonacademic audiences.

Poetic inquiry is “the use of poetry crafted from research endeavors, either before project analysis, as a project analysis, and/or poetry that is part of or that constitutes an entire research project” (Faulkner, 2020, pp. 3–4). Poetry used as qualitative research is a method of turning research interviews, transcripts, observations, personal experience, and reflections into poems or poetic forms. In this project, I use poetry as data and data analysis. The “data” for this project is based on field notes I took during the visit with my parents. As an ethnographer, I typically keep a journal of poems, notes, and reflections on events that happen as well as mundane musings (Rose, 1990). In addition, I used Facebook posts from my time in Georgia. This data served as prompts for systematic sociological introspection and reflective writing I did to fill out detailed scenes and the emotional landscape of the experience (Ellis, 2018).

I organized this piece as a series of daily menus, lyric reflections, and narrative poems that use a collage and hybrid format to detail how a family deals with political differences, identity negotiation, and crisis shown through my lyric narratives on family stories, family values, and the enactment of supportive behaviors. Some of the pieces are meant to look like recipe cards given that many of my family memories concern cooking and eating. They are also a re-storying of the family photo album and scrapbook (Faulkner, 2017a).

I began by sketching out menus and events by day using the Facebook posts and field notes. Then, I used photos that I had taken during the visit and photos that my Uncle had given me as prompts for writing reflective poems. Looking at the images helped me access memories, and the process of writing poems from the images was a kind of ekphrastic exercise. It was a back-and-forth process, writing from the photo prompted memories, looking at memories written in poems helped me access more, and so on. This writing demonstrates researcher reflexivity. Doing this writing was important for analyzing the emotional, personal, and cultural meaning of my care-giving experiences, to show how my emotions, identity and politics are imbricated. This process yielded 14 poems that I arranged in a chapbook titled *Buttered Nostalgia* to reflect the themes of food, family stories, and personal, cultural and family inheritance (Faulkner, n.d.). Next, I selected four poems to make into collage and used photoshop to manipulate the images adding the poems and recipes on top of the images. I selected seven poems to include in this piece that mirrored the themes in the daily sections (e.g., The poem, *Mother is,* became part of Thursday, March 17th because that entry was about the desire for mothering). These poems push back against familial and cultural expectations by sharing family secrets, rituals, and recipes as coping strategies for the ways that life always blows up our plans.

### Theoretical implications

I frame this work using Relational Dialectic Theory (RDT) 2.0, because of the attention paid to how dominant discourses influence family communication (Suter, 2018; Suter & Norwood, 2017). I reflect on feelings of alienation from my family based on political differences mirrored in discourses of individual freedom versus community health, science versus personal opinion, academic versus lay understandings, the tensions between wanting to be a good daughter and a good partner, and the impossible choice to stay with my parents or return to my partner and daughter. In my social network, there was support for my decision to spend as much time as I needed with my parents and apart from my daughter and spouse. I am fortunate to have empathic close friends, including a Gerontologist, with whom I can discuss the tensions between my work as a feminist professor who studies close relationships and members of my family who distrust scientific reasoning and are politically conservative. I see competing discourses of being a good daughter versus a good wife. If I were a good daughter, I would have stayed with my parents for longer, but being a good daughter competed with the marriage contract—spouse and child before anyone. Sarkisian and Gerstel (2016) argue that marriage has weakened our social ties. Single women have more social connections and are more likely to visit older parents and to offer emotional and pragmatic help. It is common for adult daughters to care for their parents, especially their mothers, when parents’ health declines, but in my case, living too far away to help daily adds to my guilt (Silverstein et al., 2006). And not having close relationships with my brothers who are more proximate to my parents precludes many of the conversations I would like to have around their health and care.

The specific entries detail the time it took me to convince my parents that COVID was real, and in fact, they constituted a high-risk group. We engaged in dialogue about evidence from the CDC about the Corona virus and information from FOX News. They listened to my expertise in understanding research and we talked about multiple different views, which mirrored my experience of being encouraged to think for myself and being allowed to have different political and social views from them and from my brothers (Feinberg et al., 2020). Other entries concern my feelings of alienation and connection to my family of origin, how focusing on common values such as the use of humor helped me reconcile feelings of dissonance and discourses of individualism, family, classism, and the public good. Estrangement from my family because of different political views is not an option, especially at a time that feels like the end of the world. Unfortunately, there is no big moment of reconciliation or series of dialogues about our differences. These tensions are still present and have become starker as the Pandemic has continued. What I see is silent acceptance of a focus on the individual as a family value and learning to live with unresolvable tensions.

## Conclusion

This work speaks to relational communication literature on social support, family narratives, and difference. It expands our understanding of what constitutes CFIC research through the use of poetic inquiry (Moore & Manning, 2019). Using poetic inquiry as a form of qualitative inquiry allowed me to tell an evocative story and critique larger cultural issues around political divides, gender and caregiving, and family values and identities (Faulkner, 2016, 2018). For me, poetry is the language of emotion, which is what made reflective narrative poetry a good tool for showing the emotional labor of caring. The use of a daily menu format demonstrates how everyday conversations engage the public-private binary and the reciprocal influences of family expectations and values, political discourse, and personal commitments. This poetic inquiry shows how public cultural discourses influence private experience and evocatively demonstrates the importance of documenting how individuals navigate difficult relational moments and competing discourses in family (cf. Berry & Patti, 2015). This work critiques the idea that family is nonvoluntary, the competing discourses of obligation and expectations competed with personal desire and understanding of being a good daughter suggesting choosing family and the concomitant roles and understandings. “All family relationships should be understood as chosen, voluntary, and less obligatory relationships, and we encourage family communication researchers to consider the harm that can stem from referring to families as nonvoluntary relationships” (Berry & Adams, 2016, p. 60). Future research should look at other family’s experiences navigating caregiving in the time of COVID.

## Notes

1. In Faulkner, S. L. (n.d.). *Buttered Nostalgia*. Unpublished Chapbook.

2. In Faulkner, S. L. (n.d.). *Buttered Nostalgia*. Unpublished Chapbook.

3. Mom’s Hot Milk Sponge. *Harpy Hybrid Review*, *2*. http://www.harpyhybridreview.org/issues/fall-2020/

4. In Faulkner, S. L. (n.d.). *Buttered Nostalgia*. Unpublished Chapbook.

5. In Faulkner, S. L. (n.d.). *Buttered Nostalgia*. Unpublished Chapbook.

6. Alice’s Buttercreams. In *International Symposium on Poetic Inquiry*.

7. Dear Alice. *Harpy Hybrid Review*, *2*. http://www.harpyhybridreview.org/issues/fall-2020/
